# What research tells us about knowledge transfer strategies to improve public health in low-income countries: a scoping review

**DOI:** 10.1007/s00038-015-0716-5

**Published:** 2015-08-23

**Authors:** Stéphanie Siron, Christian Dagenais, Valéry Ridde

**Affiliations:** Department of Psychology, University of Montreal, Pavillon Marie-Victorin, Room C355, Centre-ville Station, P.O. Box 6128, Montreal, QC H3C 3J7 Canada; School of Public Health, University of Montreal, 7101, Avenue du Parc, 3rd Floor, Montreal, QC H3N 1X9 Canada; University of Montreal Public Health Institute (IRSPUM), Montreal, Canada

**Keywords:** Scoping review, Public health, Low-income countries, Knowledge transfer, Research utilization

## Abstract

**Objectives:**

This study describes the current state of research on knowledge transfer strategies to improve public health in low-income countries, to identify the knowledge gaps on this topic.

**Methods:**

In this scoping review, a descriptive and systematic process was used to analyse, for each article retained, descriptions of research context and methods, types of knowledge transfer activities and results reported.

**Results:**

28 articles were analysed. They dealt with the evaluation of transfer strategies that employed multiple activities, mostly targeting health professionals and women with very young children. Most often these studies used quantitative designs and measurements of instrumental use with some methodological shortcomings. Results were positive and suggested recommendations for improving professional practices, knowledge and health-related behaviours. The review highlights the great diversity of transfer strategies used, strategies and many conditions for knowledge use.

**Conclusions:**

The review provides specific elements for understanding the transfer processes in low-income countries and highlights the need for systematic evaluation of the conditions for research results utilization.

**Electronic supplementary material:**

The online version of this article (doi:10.1007/s00038-015-0716-5) contains supplementary material, which is available to authorized users.

## Introduction

In its 2013 annual report, the World Health Organization (WHO) showed the extent to which scientific research can support low-income countries in achieving universal healthcare coverage (WHO 2013a). Regarding maternal and child health, the United Nations affirms that “we know what works” and “what we should do” (United Nations Secretary-General 2010). There have been countless international reports and studies explaining “what works” in fields as varied as nutrition, health services, or the fight against malaria (Allen and Gillespie 2001; Levine and What Works Working Group 2004; Peters et al. 2009). Even so, despite major advances over the past 30 years, health indicators in low-income countries remain alarming (WHO 2013b).

One challenge facing those working in public health involves the use of knowledge emanating from research. Recently, WHO African Region leaders again spoke about the “failure to apply all existing knowledge to improve people’s health” (Kebede et al. 2014). A recent review of health research among member countries of the Economic Community of the West African States (ECOWAS) urged authorities to “facilitate the use of research results to drive health policy” (Sombié et al. 2013).

Several terms are used to refer to the process leading from the production of research-based knowledge to its use by practitioners and decision makers: knowledge translation, knowledge mobilization, knowledge implementation, knowledge transfer and exchange, etc. (Graham et al. 2006; McKibbon et al. 2010). It follows that each of the terms employed to describe the phenomenon that leads produced research results into practice is defined in many different ways throughout the scientific literature. Although a multitude of definitions exist, there has yet to be a consensus identifying the most appropriate one (Pentland et al. 2011). The term knowledge transfer is, however, the most commonly employed around the world (Graham et al. 2006) and is the one used throughout this article. The Fonds de recherche du Québec—Société et culture defines knowledge transfer as “all efforts made to ensure research activities and results are known and recognized… so they can be put to use by practice settings, decision-makers and the greater public, whether the process is interactive or not” (authors’ translation) (FRQ-SC 2011, p. 9).

Use of research results can take several forms (Innvaer et al. 2002; Landry et al. 2001; Nutley et al. 2008; Weiss 1998), with the most well-documented being: (1) instrumental use (concrete changes in users’ behaviours); (2) conceptual use (changes in users’ understanding or attitudes; and (3) persuasive use (validation, support, or justification for a practice or decision).

Efforts to ensure practice settings and decision makers can benefit from scientific knowledge have led to the emergence of a new research field pursuing two objectives: (1) to evaluate the effectiveness of strategies to transfer scientific knowledge (outcomes analysis), and (2) to identify factors that might facilitate or inhibit the use of knowledge by users (process analysis).

### Outcomes analysis

Transfer processes that are considered ineffective are passive strategies used singly, such as distribution of educational materials, audiovisual materials and electronic publications (Bero et al. 1998; Corrigan et al. 2001; Dobbins et al. 2009; Grimshaw et al. 2004). Audits and feedback sessions, involvement of opinion leaders, patient-mediated interventions, conferences and use of practice guidelines produced mixed effects. Strategies shown to be effective are tailored targeted messages, combined use of multiple approaches, interactive small group meetings and computerized decision support (to help physicians with medical prescriptions). Grol and Grimshaw (2003) point out, however, that no transfer activity is effective enough to produce all types of change in all conditions. The effectiveness of strategies using multiple activities has been demonstrated (Grimshaw et al. 2004, 2006, 2012), although the literature does not report any observable relationship between number of strategies implemented and results obtained (Grimshaw et al. 2004, 2006, 2012; Walter et al. 2005).

### Process analysis

Several conditions have been identified that can influence research results use (Dagenais et al. 2013b; Denis et al. 2002; Lysenko et al. 2014; Ouimet et al. 2006), having to do with the characteristics of: (1) the transfer strategies (e.g., interaction between the transfer agent and users, user-friendliness of the activity); (2) the transfer agent (e.g., attitude toward collaboration, communications skills); (3) the users (e.g. knowledge regarding the scientific culture, understanding of the scientific results presented); (4) the organizational context (e.g., change-resistant behaviours, availability of material and financial resources); and (5) the knowledge being transferred (e.g., relevance of the topic addressed, method of presenting the information).

An analysis of the scientific literature shows it deals primarily with interventions in high-income countries, such as Australia, Canada, the United States and the United Kingdom (Bero et al. 1998; Grimshaw et al. 2004, 2006; Van Eerd et al. 2011). To our knowledge, there has been no systematic review focusing specifically on interventions in low-income countries. Yet if we are concerned about equity, this is where the needs are greatest and where populations should benefit from more effective interventions. Our objective here is to provide a synthesis of scientific results to show the current state of research on knowledge transfer in low-income countries. Specifically, this review aims to describe the main findings of the studies included, the background and research methods used and the nature of the knowledge transfer activities.

## Methods

We used the scoping review method (Arksey and O’Malley 2005), with its various stages. Unlike other systematic reviews, a scoping review does not include or exclude studies based on their research designs (Arksey and O’Malley 2005). Rather, those designs are taken into account in the data analysis. This approach allows for a comprehensive overview of all the topics, concepts and especially methodologies employed in the domain under study.

### Identification of studies

The following databases were consulted: MEDLINE and MEDLINE In-Process and Other Non-Indexed Citations, EMBASE, PsycINFO, Social Work Services Abstracts, Cochrane Database of Systematic Reviews, Cochrane Central Register of Controlled Trials, Cochrane Methodology Register, Database of Abstracts of Reviews of Effects, Health Technology Assessment Database, and NHS Economic Evaluation Database. The keywords used (English and French) were related to: (1) knowledge transfer and evidence-based practices; (2) the 36 low-income countries identified by the World Bank (2013); (3) intervention; and (4) empirical research. Table S1 in the appendix presents the strategy used to identify the studies in these different databases. In each database, our search covered the period from 1960 (when the concept of knowledge transfer first appeared) (Estabrooks et al. 2006) to May 2013.

### Selection and evaluation

Articles were selected in two stages. First, we analysed the references obtained in the identification stage based on titles and abstracts. We then examined the complete texts of the articles retained. For both stages, we used the same inclusion criteria: (1) the study was conducted in a low-income country; (2) it focused on examining knowledge transfer processes and/or their outcomes; (3) it was empirical, i.e., based on data collection and analysis; and (4) it was published in English or French. The data from the articles retained after the second stage were extracted and analysed.

### Data extraction

The analytical process consisted, first, of extracting as much information as possible to describe the studies retained. This process was based on the Grille descriptive des articles inclus dans une scoping study [Descriptive checklist for articles included in a scoping study] proposed by Malo and Robert (2011): country where the study was conducted; health issue(s) addressed; research design used; tools and instruments used to measure research use or its processes; transfer objectives pursued; transfer strategy/strategies used; and actors involved in the transfer process.

### Final data analyses

Once the data extracted, analyses of these information were performed to develop a picture of the studies’ key results, in terms of strategies used, analysis of processes and types of use measured. The data were grouped by themes and then described for qualitative analysis and quantified whenever possible. Our analysis of the research designs was guided by the Mixed Method Appraisal Tool (MMAT) developed by Pluye et al. (2011). These analyses also provided a picture of the key results of the studies.

## Results

**Fig. 1:**
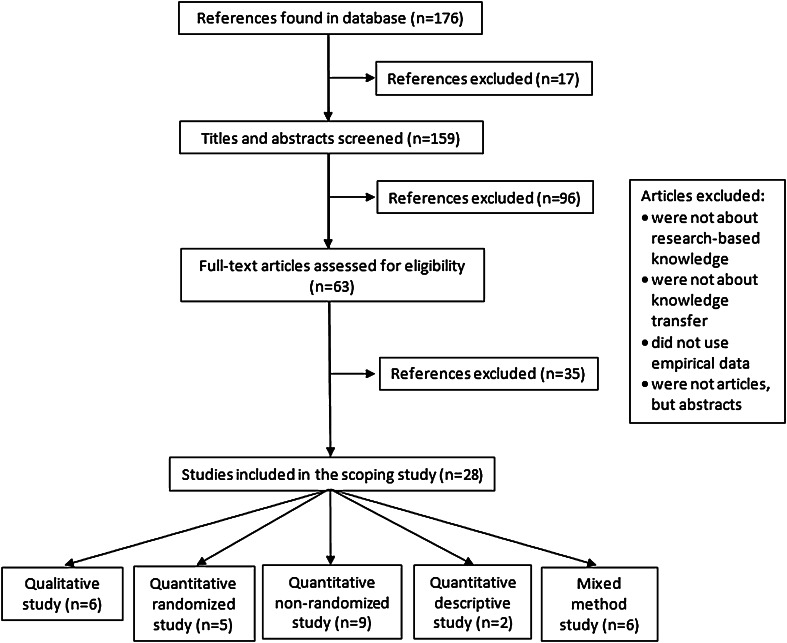
PRISMA diagram depicting the flow of information through the different phases of scoping review process about research on knowledge transfer in low-income countries

The search identified 176 potentially relevant studies. After analysis of titles and abstracts, 63 studies were retained and their full texts reviewed in depth, resulting in a final pool of 28 studies. The flow diagram has been used (see Fig. 1). Table 1 presents a complete list of these references, the key elements analysed and the results derived from each. Each of these key elements is described in more details in this section.

**Table 1:** Summary of the studies included in the review about research on knowledge transfer in low-income countries

References	Journal	Countries	Design	KT	Strategies	Use	Results
Abuya et al. (2009)	American Journal of Tropical Medicine and Hygiene	Kenya	2	O	T, Dm, Pd	I, C	Users showed a significant increase in knowledge about recommended doses and appropriate practices
Abuya et al. (2010)	PLoS One	Kenya	3	O	T, Dm	I, C	Users showed a significant increase in knowledge about recommended doses and appropriate practices
Ahmed and Zerihun (2010)	PLoS One	Uganda	3	O	T, Dm, Pd, F, R	I, C	The population studied showed limited knowledge about the results transmitted regarding the disease treatment and transmission and the preventive behaviours that should be adopted. Results on the possession and use of distributed products were not very encouraging. There were marked differences among regions
Ayieko et al. (2011)	PLoS Medicine	Kenya	2	O	T, Dm, S, G, V	I	The authors found a significant increase in the practices recommended in the practice guidelines and improved performance in terms of admission assessments
Bagamoyo College of Arts et al. (2002)	Evaluation and Program Planning	Tanzania	1	O, P	T, Pd, A, F	I, C	Artistic performances and discussions with participants helped break the silence on taboo subjects. The authors noted increases in the sense of collective responsibility, in motivation to adopt new behaviours, and in the capacity to analyse problems and find solutions. They also noted an increase in the use of recommended products and services
Blanton et al. (2010)	American Journal of Tropical Medicine and Hygiene	Kenya	3	O*	T, Dm, V, F	I, C	Users, both direct and indirect, were significantly more aware of the treatments implemented and the products available. They also showed significant increases in the knowledge transferred, the recommended health behaviours and the use of distributed products. Results were not attributable to the sociodemographic data measured
Dagenais et al. (2013b)	Global Health Promotion	Burkina Faso	1	O, P	Dm, Pd, P	I, C, P	Users appeared to be more familiar with the research process. Few consulted the published written documents. Users said they preferred workshops, even if, for these to be effective, intermediary agents had to subsequently retransmit the information into their own settings. The authors also noted changes in users’ behaviours and attitudes
Delva et al. (2010)	Tropical Medicine and International Health	Kenya	3	O*	T, Pd, R, S	I	The authors did not observe the desired improvements in health indicators. They considered that awareness raising and mobilization activities had limited effects. The frequency and timing of visits were not improved as recommended, although more prenatal services were provided
Dynes et al. (2011)	Midwifery	Bangladesh	5	O, P	T, Dm, V, F, G	I, C	Results suggested rapid integration of the program, significant improvement in performance post-training, and a high level of satisfaction with the training among participants and transfer agents
English et al. (2011)	Implementation Science	Kenya	5	O, P	T, Dm, V, G, S	I, C	The authors emphasized that the normative-educational approach and the sustained efforts exerted throughout the research project were determining factors for performance
Friend and Chertok (2009)	Public Health Nursing	Kenya	3	O*	T, Dm, V, F	I, C	Practical demonstrations significantly improved users’ skills, and the use of images was shown to be culturally effective. Overall, users’ practical knowledge was significantly improved
Gazi et al. (2005)	Journal of Health, Population and Nutrition	Bangladesh	3	O, P	T, F, S	I, C	Users demonstrated better knowledge of the services and used them more often. Their use of recommended products also increased. The results showed that local facilitators transferred knowledge, provided services and directed users toward clinics
Kaseje et al. (2010)	Global Public Health	Kenya	5	O, P	T, P	I	Results showed a marked improvement in governance committees and better practices related to the management of data and services. There was also significant improvement in the coverage of many services
Kidala et al. (2000)	Public Health Nutrition	Tanzania	2	O	T, Dm, Pd, F, L	I, C	Significant increases were seen in knowledge regarding the possession and use of distributed products and in the frequency of recommended practices
Lemay et al. (2012)	Journal of Health Communication	Malawi	5	O, P	T, Dm	I	The strategy used was costly and more effective for obtaining rapid feedback than were transitional strategies. Because of that, there was improvement noted in the quality of health services provided
Leow et al. (2011)	Journal of Surgical Education	Sierra Leone	4	O	T, Dm	I, C	Participants found the training helpful. More than 90 % of them reported that their expectations had been met, that the content was well explained and that the practical sessions were useful. They felt confident about being able to teach the skills they had learned to others in their own settings. Subsequent workshops led by the participants demonstrated the effectiveness of the strategy of developing participants’ autonomy
Libamba et al. (2007)	Bulletin of the WHO	Malawi	4	O*	T, G, S	I	Service coverage was increased
Manzi et al. (2009)	Transactions of the Royal Society of Tropical Medicine and Hygiene	Tanzania	5	O, P	T, Dm, Pd, P, S, G, V	I	After 2 months, 92 % of the health centres had implemented the use of WaterGuard. The main barrier at the start of the project was the product’s unavailability, but the supply increased as the project progressed. After 9 months, users reported they had no major problems integrating the product into their routine care practices
Nyagero et al. (2012)	Pan African Medical Journal	Kenya	3	O, P	T, Dm	I, C	Users demonstrated good practices in terms of behaviour change. These changes were associated with several sociodemographic data, in some cases significantly
Nzinga et al. (2009)	Implementation Science	Kenya	1	P	T, Dm	I	The authors identified 10 themes that could impede implementation of the transfer process
Perez et al. (2009)	BMC International Health and Human Rights	Mali	3	O, P	T, Dm, Pd, F	I, C	Results suggested the positive influence of agent users through significant increases in possession and use of distributed products and adoption of certain health behaviours. The continuation of certain non-recommended behaviours was also noted
Puchalski Ritchie et al. (2012)	International Journal of Tuberculosis and Lung Disease	Malawi	1	O, P	T, Dm, F, S	I	Local facilitators pointed out several barriers preventing them from carrying out their role and integrating knowledge into their practice. This resulted in conflicting messages, documentation errors and poor interactions with users
Shrestha et al. (2006)	Tropical Medicine & International Health	Nepal	2	O	T, Dm, F, G, V	I	Implementation of practice guidelines led to a significant reduction in multiple prescriptions. The results, although not significant, suggest that recommended practices increased and average costs of wastage decreased
Shrestha (2002)	Journal of Health, Population and Nutrition	Nepal	3	O, P	T, Dm, V, F, S	I, C	The use of visual supports was helpful and users demonstrated better knowledge after training. Group sessions allowed local facilitators to approach users and better understand their concerns, and also saved time. Six months later, significant change was noted in care-seeking behaviours
Sodhi et al. (2011)	BMC International Health and Human Rights	Malawi	5	O, P	T, Dm, F, G	I, C	Practice guidelines gave participants a greater sense of autonomy in providing appropriate health care. Participants recommended this approach because it presented several conditions that favoured implementation of the transfer process, but they also pointed out certain barriers to knowledge use
Ssengooba et al. (2011)	BMC International Health and Human Rights	Uganda	1	P	P, Pd, G, P	I	This study identified factors that facilitated absorption of knowledge and implementation of policies. Certain barriers were also pointed out
Wanyama et al. (2012)	Journal of Acquired Immune Deficiency Syndromes	Uganda	2	O, P	A, F, T	C	Users in the intervention group obtained significantly higher scores on the knowledge questionnaires. Overall, participants in the intervention group felt they had benefited from it and recommended this method. These participants and the transfer agents pointed out particular features that supported the tool
Willms et al. (2011)	Sexually Transmitted Infections	Malawi	1	O, P	P, A, L	I, C	The authors observed changes in users’ behaviours and attitudes. They describe the creation of a discussion forum, press releases and documents supporting knowledge emanating from research

Design: 1: qualitative; 2: quantitative randomized; 3: quantitative non-randomized; 4: quantitative descriptive; 5: mixed

Knowledge transfer objective: *O* outcomes, O* main objective focused on evaluation of outcomes, but certain process conditions were addressed in the discussion, *P* processes

Use: *I* instrumental, *C* conceptual, *P* persuasive

Transfer strategies: *T* training, *Dm* distribution of materials, *Pd* public dissemination, *P* partnership activities, *A* artistic activities and games, *V* visual materials, *F* local facilitator, *L* opinion leader, *S* supervision and feedback, *R* refresh sessions, *G* practice guidelines

### Source and year of publication

More than half of the studies retained had been published in the past 4 years (*n* = 17, 60.7 %) and none before 2000.

### Research setting

#### Low-income countries

The studies were conducted in 8 of the 36 low-income countries (22.2 %): Kenya (*n* = 10), Malawi (*n* = 5), Uganda (*n* = 3), Tanzania (*n* = 3), Bangladesh (*n* = 2), Nepal (*n* = 2), Burkina Faso (*n* = 1), Mali (*n* = 1) and Sierra Leone (*n* = 1).

#### Health issue(s) addressed

**Fig. 2:**
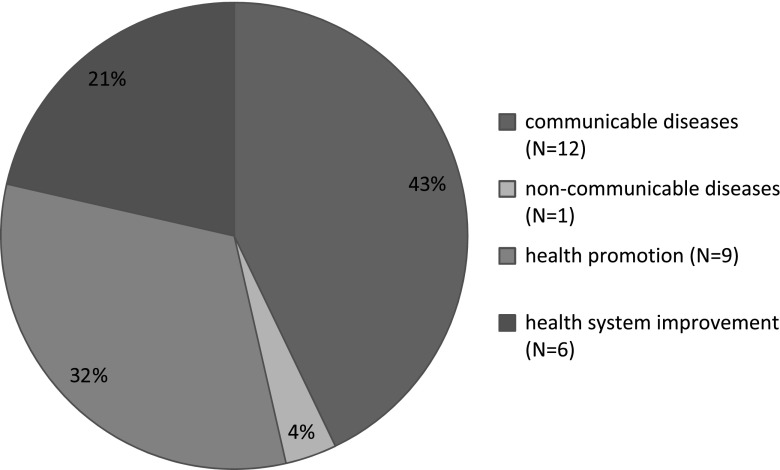
Frequencies of public health issue(s) addressed in the studies included

Four public health issues were targeted by the transfer strategies that were implemented (Fig. 2): (1) communicable diseases, such as malaria, HIV/AIDS and tuberculosis (*n* = 12, 42.9 %); (2) non-communicable diseases, such as anaemia (*n* = 1, 3.6 %); (3) health promotion, with respect to prevention measures, basic services, family planning and water treatment (*n* = 9, 32.1 %); and (4) health system improvement (*n* = 6, 21.4 %).

#### Research designs

The designs varied depending on the transfer objectives. Studies looking only at processes used exclusively qualitative designs, whereas studies that used only quantitative designs were concerned solely with outcomes analysis. Mixed designs were used in studies looking at both outcomes and processes. Of note is the fact that: only half of the studies adequately identified the limitations of their research (*n* = 14, 50 %), a few listed them summarily (*n* = 5, 17.9 %) and nearly one-third did not mention them (*n* = 9, 32.1 %) (Table 2).

**Table 2:** Analysis of research designs in studies included in the review, inspired by the Mixed Method Appraisal Tool (Pluye et al. 2011)

Qualitative designs (*n* = 6, 21.4 %)
+ Descriptions of data collection methods, formats and results took into account the research context
− Very little information was provided on the researcher’s influence on the interpretation of results
− Descriptions of participant selection or exclusion criteria were sometimes missing
Qualitative designs without random selection (*n* = 9, 32.1 %)
+ Recruitment methods helped reduce the biases associated with participant selection
+ Data were sufficiently complete to support the results
+ Measurement instruments and variables studied were generally well described
− Psychometric properties of the instruments used were poorly documented
Qualitative designs with random selection (*n* = 5, 17.9 %)
+ Sampling procedures were well explained and presented low rates of exclusion and drop-out
− Procedures for blinding were presented in only half the studies
Descriptive qualitative designs (*n* = 2, 7.1 %)
− Little information was presented on participant selection method
− No information was presented on sample representativeness
− Relevance of measurements used was mixed
Mixed designs (*n* = 6, 21.4 %)
+ Research design selected and integration of qualitative and quantitative data appear appropriate for responding to the study’s objectives
− Very little information was presented on the limitations of this integration

#### Evaluation objectives

Half of the articles (*n* = 14) attempted to both evaluate the effectiveness of the transfer strategies implemented (outcomes analysis) and understand the processes that might explain the results obtained (process analysis). The studies that concentrated only on outcomes (*n* = 11) provided very little explanation regarding effectiveness; when explanations were offered (*n* = 4), they were brief and not the main focus of the analyses.

#### Knowledge transfer strategies

A wide variety of activities—more than 30—was noted; we grouped these into seven categories (Table 3). Training programs were the most frequently used activity (*n* = 26). Most of the studies evaluated strategies that used multiple activities, whether in training programs comprising several activities (e.g., demonstration and supervision sessions, workshops), or by combining more than one strategy (e.g., distribution of materials, public dissemination, local facilitators). Other, more innovative, strategies were also highlighted, such as artistic performances and games, and the use of specially adapted materials (e.g., picture books, photographs).

**Table 3:** Knowledge transfer strategies used in the studies included

Training program (*n* = 26)
Demonstrations and practical activities; seminars and workshops; supervision and feedback; refresh sessions; peer teaching; follow-up meetings; discussion sessions
Distribution of materials (*n* = 20)
For implementation: bicycles, cellular telephones, practice guidelines and training manuals, teaching tools, wall posters in points of service
For use: medications, condoms, water containers, water purification sachets, seeds and seedlings, mosquito nets, health kits
Tailored materials: Comics, picture books, diagrams, photographs
Local facilitators (*n* = 13)
Health professionals; community members
Public dissemination (*n* = 9)
Awareness-raising activities; information sessions at points of service; health kiosks at markets; home visits; educational messages on health transmitted through the media and social networks; distribution of T-shirts with logos; scientific communications; policy briefs; press conferences
Partnership activities (*n* = 5)
Digital platform; discussion forums and exchange network for partners; workshops
Artistic performances and games (*n* = 3)
Theatre, dance, song, poetry; board games
Opinion leaders (*n* = 2)
Religious leaders; village chiefs

#### Transfer agents involved

It was usual for the same study to involve more than one type of transfer agent. Those most often mentioned were local facilitators (*n* = 14), including health professionals (*n* = 4), who received training and then, in turn, trained other less specialized professionals. There were also teachers who organized workshops with their students, and theatrical artists who gave performances and led the audience in group discussions (*n* = 4). Some local facilitators were also members of the community (*n* = 4), mothers, village chiefs or peer helpers, who were in charge of maintaining a product inventory and transmitting basic advice on the use of medications distributed. Last, several studies included instructors or trainers (*n* = 10) whose affiliations were rarely specified. The researchers (*n* = 6) represented only a small proportion of transfer agents.

#### Target users

The users most often targeted were patients/consumers (*n* = 14), primarily women (pregnant, nursing and/or with children under 5 years), but also young children and occasionally entire villages. In the category of health practitioners (*n* = 12), target users were mainly nurses, general practitioners and physicians in private practice. Political decision makers were much less often targeted as users (*n* = 3), and only one study targeted religious leaders.

### Results reported

#### Outcomes of transfer strategies

Although a few authors pointed out certain barriers to knowledge use, which will be listed in the next section, overall, the outcomes were positive. Several were significant and involved three types of knowledge use. (1) Instrumental use (*n* = 27, 96.4 %). Several positive outcomes had to do with users’ adoption of better health behaviours and increased use of essential products distributed (condoms, water purification sachets, seeds/seedlings), increases in prenatal services provided, expansion of universal coverage and appropriateness of medical prescriptions. The results also reported on closer ties created between partners and greater practice autonomy among health professionals. (2) Conceptual use (*n* = 18, 64.3 %). Several articles reported positive changes in users’ attitudes and knowledge, seen particularly in post-training questionnaires. There were also reports of improved knowledge on recommended treatments and risk factors associated with infectious diseases (e.g., HIV/AIDS, malaria), an increased sense of collective responsibility, a deeper understanding of users’ health concerns, and the emergence of a sense of engagement and responsibility among administrative managers. In some articles, users reported feeling more familiar with the research process after participating in transfer activities, while others reported a high level of satisfaction with the activities being pursued. (3) Persuasive use (*n* = 1, 3.6 %). Only one article noted that knowledge transfer strategies provided users with research results to bolster arguments.

Only three articles (Ahmed and Zerihun 2010; Delva et al. 2010; Perez et al. 2009) reported inconclusive outcomes, such as limited knowledge about the results being transmitted regarding treatment of an illness, its transmission, and prevention behaviours that should be adopted. Another one (Ssengooba et al. 2011) reported mitigated results (of two interventions, one showed inconclusive results). Only one study reported negative result: the continuation of certain non-recommended behaviour (Perez et al. 2009).

Despite the encouraging results reported in most of the articles analysed, the overwhelming use of strategies employing multiple activities made it difficult to ascertain which outcomes were specific to each activity.

#### Process analysis

The analysis identified many conditions that facilitated or impeded users’ adoption of new practices. These conditions fall into five categories (Table 4). Examples of the conditions are:

**Table 4:** Conditions for knowledge utilization identified in the studies included in the review

Conditions facilitating research results use	Conditions inhibiting research results use
A strategy that…	
Uses a participative approach	Does not involve continuing education sessions
Facilitates communication between agents and users, and among the different partners	Provides training that is too brief or too intensive
Involves local leaders	Does not promote communication among the different partners, agents and users, nor with those in charge of the implementation
Has low costs	
Makes it possible to get rapid feedback	
Is accessible (transportation) and flexible (schedule)	
Involves a method of supervision	
A transfer agent who…	
Is highly devoted and committed to the population	Is not sufficiently knowledgeable
Makes him/herself available to users	Lacks communication skills
Receives payment commensurate with the workload	Does not have the resources needed to carry out tasks (limited means of transportation)
	Is too poorly paid
Users who…	
See the benefits of using the knowledge that is transferred	Do not believe they need the knowledge being transferred
Present sufficient and accurate knowledge in relation to the evidence provided	Lack knowledge on the different aspects of the disease
Recognize the value of the evidence presented	Have financial constraints and are not ready to pay the price for essential products
	Have transportation constraints that make it difficult for them to access services
	Have beliefs and cultural practices that are in contradiction with the recommendations
	Are resistant to change
	Lack motivation
An organizational setting that…	
Facilitates the delegation of tasks among professionals	Lacks the necessary resources: space, products, and teaching materials
Fosters communication among partners, agents, and organizers of the strategies	Provides inadequate services and incomplete information and education
Value transfer activities and support agents in their activities (social acceptability)	Does not understand the community’s involvement and participation in the transfer process
Is able to receive and absorb user demands to deliver products recommended by evidence (condoms, vaccinations, primary care services, etc.)	Has a high staff turnover rate
	Does not provide the support required for supervision
	Does not enable users to access the essential products recommended by research results
Knowledge that…	
Is tailored to the context	Is too complicated to understand
Is transmitted in ways that respect users’ level of education	Is not available
Is available	

#### Favourable conditions

Creation of a rapid communication network (using SMS) and distribution of cellular telephones to allow local facilitators to obtain rapid feedback from program coordinators, to receive notifications when recommendations were changed, to share timely information with supervisors on specific medical questions or important events, and to maintain an up-to-date inventory of products distributed (Lemay et al. 2012); use of images (storytelling with scenarios) in ways that enabled people with low education to participate in problem-solving discussions (Shrestha 2002); artistic performances and audience discussions that helped break the silence on certain taboo subjects, especially HIV/AIDS illness and transmission (Bagamoyo College of Arts et al. 2002); and support to families and community recognition of women serving as depot-holders (women in the community who keep stock of commodities) (Gazi et al. 2005).

#### Unfavourable conditions

Difficulties encountered by some local facilitators in discussing family planning with participants, due to conservative cultural values that are in opposition to the recommended practices, and difficulties experienced by women in some isolated villages in trying to visit the community depot-holder or in getting to medical clinics when required for their health situation (Gazi et al. 2005); lack of adequate space and privacy to receive women and children in clinics and conduct medical interviews (Dynes et al. 2011; Puchalski Ritchie et al. 2012); lack of transportation, preventing lay health workers from conducting home visits, and lack of teaching materials, brochures and posters that would facilitate communication between front-line health workers and mothers (Puchalski Ritchie et al. 2012); and disruptions in the supply of WaterGuard, preventing users from integrating this product into their routine care practices, as recommended (Manzi et al. 2009).

## Discussion

The main findings of our synthesis are as follows: The transfer agents were primarily trainers and practitioner experts, and the users were mostly health professionals and consumers of health services (mothers, young children, village populations). All the transfer strategies studied used multiple activities, with the primary activities being training, distribution of materials and local facilitators. The studies mainly used quantitative designs, and measures of instrumental utilization were the most often used; however, the psychometric properties of the evaluation instruments were rarely reported. The primary outcomes of the evaluated strategies were increases in knowledge being transferred to health professionals and greater use by the population of recommended essential products.

### Methodological shortcomings

The articles consulted provided little or no information that would normally be used to judge methodological quality, such as on the psychometric properties of instruments, blinding procedures, the researchers’ influence on the interpretation of results and the degree of representativeness of the sample. Results obtained under those circumstances must be interpreted with caution. This situation has also been reported in systematic reviews on knowledge transfer in high-income countries (Dagenais et al. 2013a; Grimshaw et al. 2004, 2006, 2012; Van Eerd et al. 2011).

### Relevance of topics

The marked preoccupation with the treatment of communicable diseases in nursing or pregnant women and in young children coincides with these countries’ priorities (GBD 2013 Mortality and Causes of Death Collaborators 2015).

### Effective and innovative transfer strategies

The articles evaluated transfer strategies whose effectiveness has been demonstrated in other contexts. For example, many studies evaluated strategies involving multiple activities, such as training programs (Grimshaw et al. 2004, 2006, 2012) and the combined use of practice guidelines, distribution of materials and other transfer activities. These strategies have been shown to be effective in high-income countries (Corrigan et al. 2001; Grimshaw et al. 2004). The same is true with respect to the effectiveness of supervision and feedback sessions as well as of partnership activities bringing together transfer agents and potential users (Grol and Grimshaw 2003).

Some innovative strategies (see Table 3) identified in our review are rarely or never seen in studies conducted in high-income countries, and their effectiveness remains to be demonstrated. Nevertheless, they are of interest because they have many commonalities with recommendations coming out of research in high-income countries: adapting presentation methods to users’ education level, using accessible language and creating direct links between transfer agents and users (Dagenais et al. 2008; Davies et al. 2010; Gagliardi et al. 2008; Ouimet et al. 2006).

### Divergences from scientific literature from the high income countries (HIC)

The studies generally employed strategies involving multiple activities, but rarely explained this choice. Moreover, even though these strategies have been shown to be effective in low-income countries, the scientific literature does not report any clear relationship between the number of activities implemented and the outcomes achieved; the same is true for the literature on high-income countries (Grimshaw et al. 2004, 2006, 2012; Walter et al. 2005). Surprisingly, the articles analysed also present very few strategies using tailored targeted messages, which are known to be effective (Dobbins et al. 2009). These divergences may be due to the difficulty in accessing scientific results experienced by those engaged in knowledge transfer in low-income countries (Kebede et al. 2014). As well, these countries’ resource limitations may inhibit implementation of sometimes costly strategies.

Some strategies shown to be ineffective in high-income countries (Grimshaw et al. 2004) were nevertheless used in the studies reported in the articles we reviewed: awareness-raising activities, public information sessions and scientific publications. However, these were always combined with other activities that were shown to be effective or favourable to knowledge use. As such, the transfer strategies chosen in the articles were consistent with available evidence, both in terms of outcomes evaluation or process analysis.

### Foreseeable outcomes

It is not surprising that the transfer strategies reported in the articles were effective, as their characteristics are in keeping with evidence. For example, training programs, which were widely used, favour active participation (group discussions, practical activities) and are tailored to education levels (support and visual materials). Even though little data are available on the more innovative strategies used (artistic performances, games, visual materials), these are inherently favourable to knowledge use by virtue of their characteristics. The use of images has been proven to be culturally effective and appreciated by both users and transfer agents. The same is true for workshops, training programs, practical activities and group discussions, even if these require more time on all sides. The effectiveness of practice guidelines has been mixed, as reported in the scientific literature in other contexts (Grol and Grimshaw 2003). Finally, the limited outcomes of certain activities, such as consulting written documentation and awareness-raising activities—the so-called passive activities—are similar to those of activities described elsewhere (Bero et al. 1998; Grimshaw et al. 2004).

### Observable measures of use

The omnipresence of instrumental use is consistent with results reported by others (Dagenais et al. 2013a; Malo and Robert 2011). This trend may be due to the observable nature of instrumental use, which favours its measurement. The predominance of conceptual use in the articles analysed is, however, more surprising, and may be due to the strong presence of training programs, which are essentially aimed at increasing users’ knowledge. The near-absence of persuasive use has also been reported in other contexts (Dagenais et al. 2013a; Malo and Robert 2011). This may be due to the difficulty of observing and evaluating these types of outcomes, which are sometimes more diffused, or to a possible lack of instruments developed for this purpose, or to a scientific culture that is not very sensitive to this type of measure and its impacts.

### Known transfer processes

Several conditions for knowledge use reported in the articles analysed are similar to those observed in other contexts (Dagenais and Janosz 2008; Davies et al. 2010; Gagliardi et al. 2008). Examples include strategies that foster communication between agents and users, organizations that value knowledge transfer activities and have the resources to implement them, and a presentation method tailored to the audience. Organizations’ lack of resources to implement recommended transfer strategies was identified as a condition impeding knowledge use.

### Conditions for use specific to low-income countries

Examples include the frequent use of local facilitators from the community to compensate for the lack of qualified personnel. In most cases, these transfer agents are health care professionals in general or local level who receive training on a specific topic. Examples also included training programs that are sometimes very brief to minimize health professionals’ time away from their work. Also noted were careful selection of training sites and points of service to maximize accessibility, and accommodations made to facilitate transportation for the population or to reduce the geographic distance between professionals on the ground and their supervisors. Using artistic performances (theatre, songs, poems) to transmit information about diseases also showed concern for adapting to people’s beliefs and traditions (Loewenson et al. 2014). Visual tools were sometimes created to accommodate to the low education levels of the populations studied. Targeting and involving important persons was another strategy used to enhance the social acceptability of the knowledge being transferred to potential users. These strategies’ effectiveness is consistent with results from action research on factors that facilitate knowledge transfer and application (Estabrooks et al. 2003).

In these low-income countries, careful attention appears to have been given to adapting transfer strategies to their specific contexts, which is seen much less clearly in the scientific literature on other countries. Given the seriousness of the public health issues in these low-income countries and the resource constraints they face, adapting transfer strategies to these contexts seems to be a necessity, and it is encouraging to observe that many studies have made this a priority.

### Limitations of the study

Even with the systematic and retraceable process of a scoping review, the present study has certain limitations. First, only articles published in English and in French were consulted, such that articles written in the languages of certain low-income countries may have been excluded de facto. We also limited our search to scientific journals, whereas other relevant publications may have been available in the grey literature. Finally, it should be mentioned that another search strategy with slightly different inclusion criteria would have led to different research material and different conclusions.

### Conclusion

As in most studies on global health interventions or on health systems and policies, we believe it would be beneficial for researchers to examine and analyse more closely the characteristics of the context (cultural, political and economic) within which research is conducted and transfer activities implemented. It is also recommended, whenever possible, to use measurement instruments whose psychometric properties have been demonstrated. In fact, much more methodological development is required to evaluate knowledge transfer in low-income countries. Our results highlight the need for systematic evaluation of the conditions for research results use in the settings where transfer activities occur, to identify strategies that specifically target barriers inherent to the context, according to the five categories of conditions presented in Table 4. This step is likely to improve outcomes and reduce costs associated with using multiple strategies, some of which are less relevant.

While there is a need to increase the quantity and quality of research on transfer activities, there is also a strong need for more knowledge transfer interventions in low-income countries.

## Electronic supplementary material

Supplementary material 1 (PDF 190 kb)
