# Supplementary material for: What research tells us about knowledge transfer strategies to improve public health in low-income countries: a scoping review
Source: Int J Public Health. 2015 Aug 23;60(7):849–63. doi: 10.1007/s00038-015-0716-5 (PMC4636521; doi:10.1007/s00038-015-0716-5)
Supplement: Supplementary file 1 — Supplementary material 1 (PDF 190 kb) [file 38_2015_716_MOESM1_ESM.pdf]

International Journal of Public health

What research tells us about knowledge transfer strategies to improve public health in low-income countries : a scoping review

Authors : Siron, Stéphanie[1], Dagenais, Christian[1] & Ridde Valéry[2]

1: Université de Montréal, Psychology department
2 : Université de Montréal, School of public health
Corresponding author :
Christian Dagenais, Ph.D.: christian.dagenais@umontreal.ca
Department of Psychology, University of Montreal
Pavillon Marie-Victorin, room C355
P.O. Box 6128, Centre-ville Station
Montreal (Quebec) H3C 3J7
CANADA

Table 1. Search strategies used to identify studies

A. **Knowledge transfer/Knowledge use**
Synonyms searched for in titles/abstracts [.ti,ab]; Searches combined with OR

(evidence* adj1 adopt*)	(evidence* adj1 applicat*)	(evidence* adj1 disseminat*)
(evidence* adj1 exchang*)	(evidence* adj1 implement*)	(evidence* adj1 uptak*)
(evidence* adj1 utili?at*)	evidence* action*	(knowledge* adj1 adopt*)
(knowledge* adj1 applicat*)	(knowledge* adj1 disseminat*)	(knowledge* adj1 exchang*)
(knowledge* adj1 implement*)	(knowledge* adj1 mobili*)	(knowledge* adj1 transfer*)
(knowledge* adj1 translat*)	(knowledge* adj1 uptak*)	(knowledge* adj1 utili?at*)
knowledge* action*	knowledge* practic*	(research* adj1 adopt*)
(research* adj1 applicat*)	(research* adj1 disseminat*)	(research* adj1 exchang*)
(research* adj1 implement*)	(research* adj1 transfer*)	(research* adj1 translat*)
(research* adj1 uptak*)	(research* adj1 utili?at*)	research* action*
research* practic*	guideline* implement*	(innovat* adj1 adopt*)
know-do*.ti,ab.	(best* practic* adj1 adopt*	

B. Low-income countries
Low-income countries, all fields [.mp=ti, ot, ab, sh, hw, kw, tx, ct, tn, dm, mf, dv, nm, ps, rs, ui, tc, id, tm]; Searches combined with OR

Afghanistan	Bangladesh	Benin
Burkina Faso	Burundi	Cambodia
Central African Republic	Chad (Republic of)	Comoros
Dem. Republic of Congo	Eritrea	Ethiopia
Gambia	Guinea	Guinea-Bissau
Haiti	Kenya	Kyrgyzstan (Kyrgyz republic)
Liberia	Madagascar	Malawi
Mali	Mauritania	Mozambique
Myanmar	Nepal	Niger
North Korea (Dem. Republic)	Rwanda	Sierra Leone
Somalia	Tajikistan	Tanzania
Togo	Uganda	Zimbabwe

C. Interventions
Synonyms searched for in all fields [.mp=ti, ot, ab, sh, hw, kw, tx, ct, tn, dm, mf, dv, nm, ps, rs, ui, tc, id, tm]; Searches combined with OR

intervention*	program*

D. Empirical studies – only in titles and abstracts
Synonyms searched for in titles/abstracts [.ti,ab]; Searches combined with OR

study	interview	case stud*
qualitative study	quantitative study*	review*
descriptive study	data analy*	evaluation*
participant*	trial*	population
sample*	finding*	result*
met		

« Les conditions influençant l'utilisation des connaissances issues de la recherche dans les pays à faible revenus : une *scoping study* »

Par

Stéphanie Siron (SIRS30578403)

Projet d'essai
présenté en vue de l'obtention du doctorat clinique en psychologie (D.Psy.)
sous la direction de Monsieur Christian Dagenais

Lu et approuvé :

____
Signature du directeur d'essai

© Stéphanie Siron, 2011

Tables des matières

Position du problème 1

Contexte théorique 2

Utilisation des connaissances issues de la recherche 2

Transfert de connaissances 3
Stratégies de transfert des connaissances 5
Modèles conceptuels du transfert de connaissances 6

Recherche sur le transfert de connaissances 7
Conditions influençant l'utilisation des connaissances issues de la recherche 9

Interventions sociales complexes 12
État de la recherche sur le transfert de connaissances sur les interventions sociales complexes 14
Santé publique; définition et différenciation avec d'autres concepts 15

Question et objectifs de recherche 17

Démarches méthodologiques 17

Identification des études 18

Critères d'inclusion et d'exclusion 19

Extraction des données 20

Analyse des données 20

Limites de l'étude 21

Références 22

Position du problème

Au cours des dernières décennies, des efforts considérables ont été déployés afin que les connaissances issues de la recherche scientifiques profitent aux milieux de pratique et de prise de décision. Pour ce faire, une attention particulière a été portée sur les processus de transfert de connaissances par lesquels les résultats de recherche sont transformés de manière à pouvoir être utilisés. Les efforts investis dans la recherche sur le transfert de connaissance évaluent essentiellement l'efficacité des stratégies de transfert et des modèles conceptuels utilisés. Néanmoins, de plus en plus de recherches soulignent la présence de conditions individuelles et contextuelles susceptibles d'influencer l'utilisation des connaissances issues de la recherche par les milieux de pratique. En plus d'être nombreuses, ces conditions varient selon les milieux et les recherches. Dans le cadre des interventions sociales complexes en santé publique, l'étude de ces conditions est un domaine émergent et les données probantes sont encore peu nombreuses. Il devient ainsi pertinent de regrouper ces données afin de savoir ce qui a été étudié et selon quelles démarches méthodologiques privilégiées. Dans cette perspective, la question de recherche proposée est la suivante : quel est l'état actuel de la recherche sur les conditions qui influencent l'utilisation des connaissances issues de la recherche dans les interventions sociales complexes en santé publique?

Afin de comprendre les enjeux liés à un tel projet, il sera d'abord question de présenter la littérature générale entourant les connaissances issues de la recherche et le transfert de connaissances. Puis, les connaissances actuelles concernant les conditions

environnementales et individuelles seront abordées. L'état de la recherche sur le transfert de connaissances dans le cadre des interventions sociales complexes en santé publique permettra de comprendre les difficultés inhérentes à ce domaine d'étude. La dernière section exposera la méthodologie proposée pour ce projet.

Contexte théorique

Afin d'optimiser les services offerts, assurer la qualité des soins et instaurer des standards d'interventions, les milieux de pratique et de prise de décision peuvent s'appuyer sur les résultats de la recherche. Au cours des dernières décennies, des efforts considérables ont donc été déployés afin que les connaissances scientifiques profitent aux milieux de pratique et de prise de décision. Dans ce contexte, les connaissances intégrées par les professionnels et les décideurs politiques sont dites issues de la recherche (CIR) et se différencient de celles acquises sur la base de l'expérience et donc dites issues de la pratique. Ceci a favorisé l'émergence du mouvement *Evidence-Based Pratices*, ou les pratiques fondées sur les données probantes. Cette tendance apparaît d'abord en médecine au début des années 1980, puis gagne ensuite le domaine de la santé publique et de la politique (Woolf & Atkins, 2001). Les pratiques fondées sur les données probantes sont maintenant conseillées par plusieurs ordres professionnels comme ligne de conduite.

Utilisation des connaissances issues de la recherche

L'utilisation des connaissances issues de la recherche dans la pratique peut prendre plusieurs formes, dont les plus courantes sont le type *instrumental*, *conceptuel* et

symbolique (Huberman, 1993; Weiss, 1998). L'utilisation instrumentale signifie que l'application des connaissances se fait de façon concrète, entraînant des changements dans les comportements et les pratiques des intervenants, des administrateurs ou des décideurs du milieu. L'utilisation conceptuelle quant à elle, s'observe lorsque les connaissances transférées entraînent des changements dans la compréhension et l'attitude des professionnels et des décideurs politiques concernant leur pratique (Innvaer, Vist, Trommald, & Oxman, 2002). Enfin, lorsque les connaissances permettent de valider une pratique, supporter des actions politiques ou justifier des priorités financières, il est question d'une utilisation de type symbolique (Landry, Amara, & Lamari, 1998; Nutley, Jung, & Walter, 2008).

Les connaissances produites par la recherche ne sont toutefois pas toutes intégrées aux pratiques des professionnels et des décideurs. Denis, Hébert, Langlay, Lozeau et Trottier (2002) parlent du phénomène de *sous-adoption* des connaissances lorsque les pratiques soutenues par des données probantes sont peu utilisées par les milieux. Inversement, le phénomène de *sur-adoption* des connaissances opère lorsque des innovations sont rapidement mise en place alors qu'aucune donnée probante ne permet d'en justifier l'utilisation efficace et sécuritaire. La présence de telles divergences reflètent bien la complexité des processus par lesquels les connaissances issues de la recherche sont transférées des milieux de recherche aux milieux de pratique et de prise de décision. L'étude des processus de transfert des connaissances permet de mieux comprendre l'ampleur des enjeux liés à un tel sujet.

Transfert de connaissances; définition et concepts associés

Un grand nombre de termes sont employés dans la littérature pour désigner le processus conduisant de la production des résultats de la recherche à leur utilisation par les milieux de pratique et de prise de décision (Graham et al., 2006; McKibbon et al., 2010) : échange de connaissances, transfert de connaissances, dissémination, application des connaissances, diffusion des connaissances et gestions des connaissances, pour n'en nommer que quelques-uns. Les Instituts de recherche en santé du Canada (IRSC) ont définit le transfert des connaissances comme étant le « processus par lequel les connaissances passent d'une partie à une autre dans un objectif d'élaboration ou d'amélioration de produits, de services ou de pratiques » (IRSC, 2011). Ces deux parties constituent d'une part celle qui dispose de la connaissance et qui la transmet, c'est-à-dire l'agent de transfert, et d'autre part celle qui la reçoit, aussi nommé l'utilisateur potentiel des connaissances. Parce qu'il est le principal producteur des connaissances issues de la recherche, le chercheur est souvent considéré comme le seul agent de transfert possible duquel les connaissances scientifiques sont transmises. Cependant, on fait de plus en plus appel à des intermédiaires entre les chercheurs et les utilisateurs et ces tiers collaborateurs jouent de plus en plus le rôle d'agent de transfert. Leurs tâches consistent entre autre à sélectionner, transformer et transmettre les connaissances issues de la recherche, ou encore à assurer la diffusion des résultats de recherche à grande échelle (Dobbins et al., 2009; Nutley, 2010). Les utilisateurs quant à eux représentent un éventail de professionnels et de décideurs politiques et se retrouvent dans des milieux tous aussi variés que des centres communautaires, des centres hospitaliers, des organismes à but non lucratifs et des institutions gouvernementales.

Le transfert de connaissances est un processus ayant pour objectif de favoriser l'intégration des connaissances issues de la recherche par les milieux de pratique et de décision. Afin de transférer les connaissances issues de la recherche, *différentes stratégies* peuvent être utilisées. De plus, l'implantation des activités de transfert diffère selon le *modèle conceptuel* choisi. Les sections qui suivent abordent ces deux sujets plus en détails.

Stratégies de transfert des connaissances. Les stratégies de transfert sont nombreuses et se présentent sous des formes variées. Les chercheurs les regroupent généralement en trois grandes catégories passives, actives et mixtes, selon le niveau d'investissement qu'elles exigent tant de celui qui transmet les connaissances que de celui qui les reçoit. Les stratégies *passives* correspondent au niveau d'investissement minimal de part et d'autre. Ces stratégies incluent notamment l'affichage, la distribution d'un résumé, la suggestion de lecture et la création de bases de données électroniques (Institute for Work & Health, 2011). Les stratégies *actives* sont celles qui demandent davantage l'implication de la part des deux parties, agents et utilisateurs (Dobbins, Rosenbaum, Plews, Law, & Fysh, 2007). Des agents de transfert peuvent par exemple répondre aux besoins spécifiques des utilisateurs par la création de synthèses de données « sur mesure » (traduction libre de *tailored and targeted message*). De leur côté, les utilisateurs peuvent participer à des ateliers de travail (*workshop*) afin d'acquérir les nouvelles connaissances et les intégrer à leur pratique. Finalement, les stratégies *mixtes* sont celles qui combinent à la fois les stratégies passives et actives. Ces stratégies sont habituellement mise en place par des agents intermédiaires, dont notamment des courtiers

de connaissance, dont le rôle peut impliquer de diffuser les connaissances, d'adapter la présentation des résultats, d'évaluer les besoins du milieu et de participer à la rédaction de politiques locales (Dobbins et al., 2009; Nutley, 2010). Dans tous les cas, les agents intermédiaires ne produisent pas de nouvelles connaissances, mais ils facilitent les processus de transfert et surtout, favorisent l'utilisation des CIR.

Modèles conceptuels du transfert de connaissances. Plusieurs dizaines de modèles ont été proposés dans le but d'expliquer le processus de transfert de connaissances (Estabrooks, Thompson, Lovely, & Hofmeyer, 2006; Graham et al., 2006; Nutley et al., 2008; Speller, Wimbush, & Morgan, 2005; Wilson, Petticrew, Calnan, & Nazareth, 2010). Trois principaux types de modèles ressortent de la littérature : *1)* les modèles descendants de diffusion chercheur-utilisateur (*top-down*), *2)* les modèles axés sur la résolution de problème et *3)* les modèles coopératifs. Le premier type de modèle réfère au transfert de connaissances comme un processus de diffusion unidirectionnel allant du chercheur vers l'utilisateur. C'est entre autre le cas du modèle « *science push* » de Denis, Lehoux, et Champagne (2004). Selon cette perspective, les objets de recherche sont principalement déterminés par les intérêts des chercheurs et les démarches scientifiques sont menées indépendamment des besoins du milieu. L'intégration des CIR repose alors sur les compétences de l'agent de transfert à disséminer ses résultats à l'utilisateur de manière adéquate. Le rôle de l'utilisateur est ici de recevoir passivement les connaissances qui lui sont transmises (Denis, Lehoux, & Champagne, 2004; Gélinas et Pilon; 1994; Nutley et al., 2008; Speller, Wimbush, & Morgan, 2005). D'autres modèles, dont le modèle « *demand pull* » (Denis, Lehoux, & Champagne; 2004),

proposent à l’inverse de concevoir le transfert de connaissances comme un moyen de mettre la science au service de la pratique en partant des utilisateurs vers les chercheurs. Dans cette perspective, les objets de recherche sont déterminés par les préoccupations des milieux. Elle exige une participation active des utilisateurs, puisque ceux-ci doivent identifier leurs besoins avant de les transmettre aux chercheurs. Dans ces modèles, le rôle du chercheur est d’offrir des connaissances accessibles, mais surtout pertinentes et en lien avec les besoins exprimés par les milieux (Denis, Lehoux, & Champagne, 2004; Nutley et al., 2008; Speller, Wimbush, & Morgan, 2005). Ces deux premiers modèles suivent une progression unidirectionnelle. Dès le début des années 1990, Gélinas et Pilon (1994) mettaient en opposition ces modèles dits « linéaires » avec les modèles « coopératifs » qui favorisent plutôt les interactions entre les deux partis. Ce type de modèle met l'accent sur une dynamique collaborative entre les chercheurs et les milieux utilisateurs et ce, tout au long du processus de recherche. Dans ces modèles, les connaissances pratiques de l’utilisateur sont associées aux compétences scientifiques du chercheur afin de réaliser un projet commun. Les questions de recherche étudiées dans ce contexte combinent les problématiques vécues par les utilisateurs et les intérêts des chercheurs.

Recherche sur le transfert de connaissances

L'intérêt d’évaluer l’utilisation des connaissances issues de la recherche par les milieux de pratique et de prise de décision apparait dans les années 1990. Une des premières observations fut alors de constater la sous-utilisation des CIR (Feldman, 1999; Rich 1997; Weiss 1988). Par exemple, des chercheurs américains ont démontré que 30 à 40% des patients ne recevaient pas les traitements recommandés par les résultats des

recherches et que 20 à 25 % recevaient même des services qui n'étaient pas nécessaires, voir potentiellement dangereux (Grol, 2001; Grol & Grimshaw, 2003; McGlynn et al., 2003; Schuster, McGlynn, & Brook, 1998). Afin de mieux comprendre les processus menant à l'utilisation des connaissances scientifiques, les chercheurs se sont penchés sur l'étude du transfert des connaissances et de plus en plus d'organismes subventionnaires canadiens financent la recherche sur le sujet.

La recherche sur le transfert de connaissances et l'utilisation des connaissances issues de la recherche porte essentiellement sur l'efficacité des types de stratégies de transfert (Bero et al., 1998; Dobbins, et al., 2009; Dobbins et al., 2007; Grol & Grimshaw, 2003) et les modèles conceptuels (Jacobson et al., 2003; Nutley et al., 2009; Wilson et al., 2010). Une récente étude dans le domaine de l'éducation suggère toutefois que la fréquence actuelle d'utilisation des connaissances scientifiques reste encore faible. Ainsi, Abrami et ses collègues (2010) soulignent que même si 65% à 80% des acteurs du milieu scolaire affirment avoir déjà consulté une des sources de CIR, ceux-ci ne s'y référeraient qu'une à deux fois par année en moyenne. Par ailleurs, les recherches menées sur les processus de transfert de connaissances permettent d'identifier plusieurs conditions contextuelles et individuelles susceptibles d'influencer l'utilisation des connaissances scientifiques par les milieux de pratique et de prise de décision (Dagenais, Janosz, Abrami, Bernard, & Lysenko., 2008; Denis et al., 2002; Ouimet, Landry, Amara, & Belkhodja, 2006). Ces conditions méritent une attention particulière dans le cadre de ce projet. La prochaine section porte sur l'énumération et la description de celles-ci.

Conditions influençant l’utilisation des connaissances issues de la recherche. Afin de mieux cerner, comprendre et étudier les conditions qui influencent l’utilisation des connaissances issues de la recherche, certains auteurs proposent de les regrouper (Dagenais 2010; Ouimet et al., 2006). Les sections suivantes présentent une classification en cinq catégories, selon les caractéristiques auxquelles les conditions sont associées :*1)* caractéristiques des stratégies de transfert; *2)* caractéristiques de l'agent de transfert; *3)* caractéristiques du contexte organisationnel de l’utilisateur et de l'agent de transfert; *4)* caractéristiques de la connaissance transférée; et *5)* caractéristiques de l’utilisateur.

Les conditions associées aux caractéristiques des stratégies de transfert. Concernant les conditions de cette catégorie, nommons entre autre le niveau de rapprochement encouragé entre l'agent de transfert et les utilisateurs. Certains auteurs mentionnent à cet effet qu’une interaction directe et dynamique (Dagenais et al., 2008; Ouimet et al., 2006) ainsi que l’implication des utilisateurs potentiels dans les activités de recherche (Estabrooke et al., 2003) sont des conditions qui facilitent le processus de transfert de connaissances et favorisent l’utilisation des connaissances issues de la recherche. Il est également favorables que les rôles de chacun, tant agent de transfert qu’utilisateur, soient clairement définit et que la relation soit basée sur la confiance mutuelle, facilitant ainsi la résolution de problème le temps venu (Dagenais & Janosz, 2008).

Les conditions associées aux caractéristiques de l'agent de transfert. Une attitude positive envers la collaboration et la présence de bonnes habiletés de communication, sont quelques exemples des conditions favorables associées à l'agent de transfert, qu’il soit chercheur ou agent intermédiaire (Dagenais & Janosz, 2008). Or, plusieurs

chercheurs interrogés par Dagenais et Janosz (2008), précisent avoir besoin de soutien spécifique en ce qui concerne le volet « communication » des connaissances. Ceux-ci proposent notamment l’embauche d'experts dédiés aux tâches de transfert telles que la vulgarisation scientifique, la préparation des présentations et l'adaptation du langage scientifique selon le public cible. . La connaissance des milieux utilisateurs par les chercheurs leur permettrait aussi d’être davantage conscients de la culture de l’organisation, des conditions contraignantes et des intérêts propres à ces milieux (Dagenais & Janosz, 2008). Dans cette situation, le chercheur pourrait s'adapter aux demandes particulières du milieu, ce qui favoriserait un transfert de connaissances plus efficace.

Les conditions associées aux caractéristiques l’utilisateur. Au niveau des conditions de cette catégorie, Dagenais et al. (2008) mentionnent l’opinion des utilisateurs face à la recherche ainsi que leurs expériences antérieures dans un processus de transfert de connaissances. Par exemple, la méconnaissance de la culture scientifique et des processus de recherche par les utilisateurs peut être associée à la présence d'attentes irréalistes, des difficultés de compréhension concernant les résultats de recherche présentés et la difficulté à exprimer clairement leurs préoccupations (Dagenais & Janosz 2008). Ces conditions sont susceptibles d'entraver les processus de transfert de connaissances mis en place, mais ne sont toutefois pas insurmontables et peuvent s’estomper au fur et à mesure que les parties collaborent sur des projets communs (Dagenais et Janosz, 2008).

Les conditions associées aux caractéristiques du contexte organisationnel de l’utilisateur et de l'agent de transfert. Un contexte organisationnel peu favorable à

l'intégration de nouvelles connaissances et qui favorise des comportements de résistance aux changements de pratiques nuit à l'utilisation des connaissances issues de la recherche (Dobbins et al., 2007; Gagliardi et al., 2008; Innvaer et al., 2002). De plus, certains utilisateurs interrogés par Dagenais et Janosz (2008) mentionnent que la recherche d'informations et la participation à des projets en collaboration sont des tâches qui prennent du temps, mais qu'elles ne sont pas considérées dans leur charge de travail régulière. Ils soulèvent ainsi la difficulté à intégrer les activités de transfert dans leurs tâches quotidiennes. Ce même phénomène est également mentionné par certains des chercheurs interrogés par Dagenais et Janosz (2008). Ceux-ci soulèvent en effet la difficulté de faire reconnaitre les activités de transfert au même titre que les tâches administratives et celles liées à la recherche et à l'enseignement, davantage favorisées par le milieu universitaire et les organismes subventionnaires. Ils déplorent entre autre le manque de ressources matérielles, financières, techniques et logistiques qui leurs sont allouées pour implanter des activités de transfert (Dagenais & Janosz, 2008).

Les conditions associées aux caractéristiques de la connaissance transférée. La pertinence du sujet traité, l'adéquation de celui-ci avec les besoins des utilisateurs et la perception d'impacts positifs sur leur clientèle sont des conditions qui favorisent l'intégration de la connaissance transférée par les utilisateurs (Dagenais et al., 2008; Gagliardi et al., 2008). L'adaptation du mode de présentation des résultats favorise également l'utilisation des connaissances issues de la recherche, dont la création de synthèses (Bowen & Martens, 2005), l'emploi d'une stratégie conviviale (Dobbins et al., 2007) et l'utilisation d'un langage facile à comprendre (Gagliardi et al., 2008; Nutley, 2010).

Bien que de nombreuses conditions aient pu être identifiées, celles-ci varient d’un milieu à un autre (Ouimet et al., 2006) et d’une étude à l’autre (Dagenais et al., 2008), rendant difficile l’évaluation réelle de l'impact de chacune dans le processus de transfert de connaissances. Un manque de connaissances et de précision sur le sujet pourrait ainsi entraîner des conclusions erronées et de fausses généralisations. Afin d’éviter que cela se produise, certains chercheurs recommandent de mieux documenter les conditions environnementales et individuelles susceptibles d’influencer le processus de transfert de connaissances (Egan et al., 2009).

Interventions sociales complexes

Peu de chercheurs définissent clairement la notion d’une intervention sociale complexe (Craig et al., 2008; Egan et al., 2009; Pawson et al., 2005). Dans l'ensemble, ces interventions se déroulent dans un contexte social, plutôt qu’individuel et mettent en jeu plusieurs acteurs dans un processus d’intervention non-linéaire. Face à un problème tel que le diabète ou l’obésité morbide, un patient pourrait recevoir des traitements médicaux, probablement adaptés à sa condition particulière et ne s’appliquant qu’à lui. Or, un programme de sensibilisation concernant l'importance de la bonne l'alimentation et l'activité physique chez les jeunes du secondaire traiterait du même sujet et permettrait de rejoindre un plus grand nombre de personnes. À cause de sa portée sociale et de l’implication des multiples acteurs (enseignants, directeurs d’écoles, gestionnaires de commissions scolaires, infirmières, etc…) cet exemple représente une intervention sociale complexe. Pawson et ses collègues (2005) définissent plus spécifiquement les

interventions sociales complexes selon sept caractéristiques. *1)* Les interventions sociales complexes sont basées sur des hypothèses. Tel qu'expliqué par Pawson et ses collègues (2005), l'implantation d'une intervention sociale complexe se base sur la présomption qu'elle apportera davantage de résultats que la précédente, jugée inappropriée ou trop peu performante. Cette logique nécessitera d'évaluer les résultats de l'implantation afin de confirmer, ou infirmer, l'hypothèse émise. *2)* Les interventions sociales complexes sont actives, c'est-à-dire qu'elles impliquent l'action de plusieurs acteurs du milieu. Or, l'action de chaque acteur est influencée par son raisonnement et ses choix personnels. À la différence des recherches traditionnelles qui tentent d'éliminer les facteurs personnels au profit d'une expérimentation contrôlée randomisée, les auteurs soulignent qu'il est important de considérer la subjectivité individuelle dans l'analyse des résultats d'une intervention sociale complexe. *3)* Les interventions sociales complexes consistent en une chaîne de processus. Que ce soit à l'étape de la conceptualisation, de la gestion des ressources ou de l'application clinique, plusieurs professionnels auront à penser ou mettre en pratique l'intervention en question. Pour les auteurs, le succès final dépend ainsi du cumul des succès réalisés tout au long de ce processus. *4)* L'implantation d'une intervention sociale complexe n'est pas linéaire, utilisant tant le modèle ascendant (*bottom-up*) que descendant (*top-down*). Pour les auteurs, l'orientation du modèle utilisé dépend principalement de l'influence et du pouvoir de chacune des parties impliquées. De plus, la possibilité de revenir sur des étapes antérieures de l'implantation permet d'ajuster l'intervention sociale complexe afin qu'elle réponde adéquatement aux besoins du milieu d'implantation (Craig et al., 2008). *5)* Les interventions sociales complexes sont sensibles au contexte social dans lequel elles se déroulent. Rares sont en effet les

milieux présentant des caractéristiques identiques, que ce soit en terme de ressources disponibles, de structure organisationnelle ou encore au niveau des relations interpersonnelles entre les employés. En d'autres mots, l'application d'une intervention sociale complexe doit être contextualisée et ne peut se faire de manière standardisée. *6)* Les interventions sociales complexes sont sujettes aux changements. Pour reprendre l'exemple des auteurs, un même programme d'éducation à la sexualité est susceptible d'être présenté très différemment s'il est présenté dans un couvent pour filles ou dans une école mixte. En s'adaptant aux partialités du milieu, les interventions sociales complexes deviennent le produit (unique) du contexte dans lequel elles s'inscrivent. *7)* Les interventions sociales complexes sont des systèmes ouverts apprenant, c'est-à-dire qu'elles modifient les conditions initiales du contexte d'implantation. Les apprentissages réalisés par les acteurs du milieu tout au long du processus ont en effet pour conséquences de modifier leur réceptivité et influencer leur raisonnement face à l'application de l'intervention. Selon les auteurs, l'évolution du contexte telle qu'elle apparaît dans l'implantation d'une intervention sociale complexe a la particularité d'entraîner des effets inattendus à long terme.

État de la recherche sur le TC dans le cadre des interventions sociales complexes. Comme c'est le cas dans d'autres domaines, la recherche sur le transfert des connaissances dans le cadre des interventions sociales complexes présente peu de données probantes. Les chercheurs expliquent cette situation entre autre par la difficulté d'utiliser les méthodes de recherches traditionnelles pour évaluer les problématiques sociales touchés par les interventions sociales complexes (Craig et al., 2008; Pawson et

al., 2005). D’un côté les méthodes traditionnelles tentent de mesurer le niveau d’efficacité d’un programme en se basant sur la contrôlabilité des variables et la standardisation des protocoles de recherche. De l’autre, les interventions sociales complexes se présentent sous des formes diverses, impliquent de multiples acteurs, s’ajustent aux particularités du milieu et suivent un processus d’implantation itératif (Denis et al., 2002). Ne pouvant tenir compte de toute la complexité des interventions sociales complexes, il semble ainsi que les méthodes traditionnelles appliquées dans ce cadre particulier produisent des résultats souvent contradictoires ou peu concluant (Pawson et al., 2005).

Santé publique; définition et différenciation avec d'autres concepts. Au-delà des processus de promotion, de prévention et de protection en matière de pratiques sanitaires, la santé publique vise « l'amélioration de l’état de santé des individus, pris dans leur ensemble et non individuellement » (Ridde, 2007). Afin de bien comprendre les limites des interventions appliquées en santé publique, celles-ci doivent être différenciée des pratiques en *promotion de la santé* et en *santé communautaire*. Dans un premier temps, la définition de la santé publique est dite *positive* car elle vise un objectif d'amélioration. Elle se différencie ainsi de la promotion de la santé dont les pratiques visent un objectif négatif en matière de santé par la réduction des inégalités sociales. Dans un deuxième temps, les pratiques en santé publique s’appuient essentiellement sur les données probantes et s’adressent généralement aux institutions gouvernementales telles que les systèmes de santé et de l’éducation. Les processus technocratiques de la santé publique la différencient ainsi de la santé communautaire dont la participation de la

population est au centre des interventions (Ridde, 2007). Fondés sur des hypothèses d'amélioration de la santé individuelle, les objectifs des interventions sociales complexes en santé publique visent ainsi la promotion, la prévention et la protection de la santé des populations et des communautés. De plus, ces interventions se caractérisent par la participation de nombreux acteurs locaux, sont influencées par le contexte social et s'ajustent en fonction des particularités du milieu d'implantation (Craig et al., 2008; Egan et al., 2009; Pawson et al., 2005) .

L'analyse des écrits scientifiques sur le transfert de connaissances permet deux constats. D'abord, les recherches en santé publique ne portent pas sur les conditions contextuelles et individuelles susceptibles d'influencer l'utilisation des connaissances issues de la recherche. Les résultats obtenus sur ce sujet proviennent notamment du domaine de l'éducation (Dagenais & Janosz, 2008) et des sciences infirmières (Estabrooke et al., 2003) et de la santé ont étudié les facteurs, réels ou perçus, qui facilitent l'utilisation des connaissances issues de la recherche chez les décideurs (Ouimet et al., 2006; Gagliardi et al., 2008; Innvaer et al., 2002) ou qui sont associés aux caractéristiques du contexte organisationnel (Bowen & Marten, 2005). Ensuite, parmi les revues systématiques et recension des écrits sur le transfert des connaissances issues de la recherche (Bero et al., 1998, Grimshaw et al., 2004), aucune ne porte sur les interventions sociales complexes en santé publique.

Dans un contexte émergent, où les définitions et les termes utilisés sont variables, les sujets étudiés complexes et les données probantes encore peu nombreuses, il importe de

réaliser un portrait des connaissances disponibles afin de connaître l'état de la recherche actuelle et orienter judicieusement les prochaines études.

Question et objectifs de recherche

La question de recherche de ce projet d'essai doctoral peut être posée de la manière suivante : Quel est l'état actuel de la recherche sur les conditions qui influencent l'utilisation des connaissances issues de la recherche dans les interventions sociales complexes en santé publique? Pour ce faire, le présent projet vise à répertorier toutes les études pertinentes traitant du sujet et ce, incluant tous les type de devis de recherche utilisés. Une des façons de procéder à ce type de démarche méthodologique particulière a récemment été définie par Arksey et O'Malley (2005) et se nomme « *scoping study* ».

Démarches méthodologiques

La *scoping study* est une démarche exploratoire, qualitative et descriptive. Cette démarche méthodologique, que les IRSC ont traduits par *étude de portée,* suit des étapes définies et systématiques : *1)* définir la question de recherche, *2)* identifier les études pertinentes, *3)* sélectionner les études, *4)* regrouper les données et *5)* synthétiser les résultats (Arksey & O'Malley, 2005). Une des particularités de la *scoping study* est qu'elle ne tient pas compte des devis utilisés par les études comme critères d'inclusion et d'exclusion. Cette caractéristique permet de faire un tour d'horizon complet des objets de recherches, des concepts et surtout des méthodologies employées dans le domaine de

recherche étudié. La différenciation des devis de recherche des études consultées s'effectue plus tard dans l'analyse des données (Arksey & O'Malley, 2005). La *scoping study* se différencie ainsi de la revue systématique, laquelle porte sur l'étude d'une question bien précise en recherchant un devis de recherche particulier.

Identification des études

Plusieurs stratégies d'identification des études seront utilisées. Les principales bases de données qui seront utilisées et qui sont susceptibles de présenter un maximum d'articles pertinents pour la présente étude sont les suivantes: *MEDLINE and MEDLINE In-Process and Other Non-Indexed Citations*, *EMBASE*, *CINAHL*, *PsycINFO*, *EconLit*, *Social Services Abstracts*, *Social Policy and Practice*, *Cochrane Database of Systematic Reviews*, *Cochrane Central Register of Controlled Trials*, *Cochrane Methodology Register*, *Database of Abstracts of Reviews of Effects*, *Health Technology Assessment Data*base, *NHS Economic Evaluation Database*. La base de données de l'équipe de recherche dirigée par le Dr. Dagenais, spécialisée sur les recherches en TC, sera également consultée pour la recension des articles. Des recherches manuelles seront également effectuées auprès des journaux électroniques qui ne sont pas indexés aux bases de données précédentes, dont par exemple *Global Health Promotion, BMC Public Health*, *Evaluation and Program Planning*, *Health Promotion Practice.* Finalement, la méthode « boule-de-neige » permettra de compléter la liste des références consultée. Cette méthode consiste à trouver des articles à partir de la liste de référence d'une première étude puis, à répéter l'opération pour chaque nouvel article sélectionné jusqu'à ce que les références dans le domaine soient épuisées, c'est-à-dire, jusqu'à ce qu'aucune

nouvelle étude ne puisse être citée. Afin d'empêcher la duplication d’un même article, les titres et résumés de chacun seront importés électroniquement et gérés par le logiciel bibliographique EndNote.

Critère d’inclusion/exclusion

Dans un premier temps, les articles seront sélectionnés selon leur titre et les données de leur résumé, et devront avoir pour sujet principal le TC en santé publique. Utilisant des critères d’inclusion plutôt larges, préférant à cette étape davantage de faux positifs que l’inverse, les articles sélectionnés devront *1)* porter sur les ISC et les processus du TC (stratégies de TC ou conditions influençant) ou ses effets, *2)* être disponibles en anglais ou en français et *3)* avoir été publiés à partir de 1960. Le choix de cette date correspond à la décennie durant laquelle le concept de transfert de connaissances fait son apparition dans la documentation scientifique (Estabrooke et al., 2006). Le premier tri utilisera l’entrée d’une combinaison de mots clé dans les moteurs de recherches des bases de données électroniques sélectionnées. Les mot-clé servant à la recherche des articles feront l’objet d’un consensus avec le superviseur et seront tirés principalement de la littérature ainsi que des thésaurus des bases de données. Des critères d'exclusion seront aussi appliqués; les articles seront exclus des analyses s’ils : *1)* portent sur les interventions cliniques, *2)* ne sont pas rédigés en anglais ou en français, *3)* ont été publiés avant 1960. Tous les articles sélectionnés à la première étape seront étudiés à nouveau mais cette fois-ci dans leur intégralité, utilisant les mêmes critères d’inclusion et d’exclusion que pour l’étape précédente.

Extraction des données

Un premier type d'extraction permettra d'obtenir un minimum d'information pour « identifier » les études sélectionnées, soit les auteurs, l'année et le lieu de publication, le type d'échantillon, les types d'utilisateurs, le domaine d'étude. Les données concernant la description générale de l'article ainsi que sa méthodologie seront extraites des études sélectionnées. En s'inspirant de la « grille descriptive des articles inclus dans une *scoping study* » proposée par Malo et Robert (2011), il sera possible de regrouper les informations suivantes : les objectifs de la recherche, l'objet de TC, les conditions environnementales et individuelles influençant l'utilisation des connaissances issues de la recherche, les fondements théoriques, le domaine de santé publique, la stratégie d'échantillonnage, échantillon, instruments de mesure (si données quantitatives), techniques de recherche (si données qualitatives), outils d'analyse, principaux résultats. L'attention particulière que portent Malo et Robert (2011) aux stratégies de TC implantées ainsi qu'à leur cible sera ainsi remplacée par les conditions qui influencent l'utilisation des CIR. Finalement, la grille d'analyse de Pluye et al. (2011), intitulé *Mixed Method for Appraisal Tool* (MMAT), permettra d'évaluer la qualité des études selon 5 types de devis : qualitatif, quantitatif contrôlé avec sélection aléatoire, quantitatif sans sélection aléatoire, observationnel descriptif et mixte.

Analyse des données

Les analyses permettront de répondre aux objectifs de la présente démarche, soit d'examiner l'étendue et la nature des recherches portant sur les conditions influençant l'utilisation des connaissances issues de la recherche sur les interventions sociales

complexes en santé`publique, et de déterminer la valeur scientifique de celles-ci. Pour ce faire, les analyses regrouperont les études sélectionnées selon le type de devis utilisé et mettra ses résultats en lien avec les conditions identifiées par les études sélectionnées. Le domaine spécifique d'application de l'intervention sociale complexe étudié (santé internationale, santé des communautaire, santé mentale, etc.), sera aussi être décrit par ces analyses. En identifiant les domaines les moins étudiés ou ceux dont la qualité scientifique ne permet pas de tirer des conclusions solides, les analyses permettront de proposer des pistes pour les recherches futures sur le sujet.

Limites de l'étude

Malgré la systématisation des étapes de la *scoping study*, celle-ci présente certaines limites. La principale se retrouve au niveau de la barrière langagière; il est en effet impossible de consulter tous les articles rédigés dans toutes les langues. Il est donc très probable que certains articles pertinents pour le présent projet ne soient rédigés ou traduits ni en anglais ni en français et donc, ne seront consultés. De plus, l'absence d'un second avis au moment de la sélection des articles entraîne un biais subjectif lié au chercheur. L'absence d'un accord inter-juge nuit ainsi à l'objectivité de la validation de cette sélection.

Références

Abrami, P.C., Lysenko, L., Janosz, M., Bernard, R.M., & Dagenais, C. (2010). L'utilisation des connaissances issues de la recherché par les écoles. In M. Janosz, J. Bélanger, C. Dagenais, F. Bowen & P.C. Abrami (Eds.). *Rapport final d'évaluation de la mstratégies d'intervention Agir autrement. Volume 1 – La mise en oeuvre de la Stratégies*. Montréal, Qc : Groupe de recherche sur les environnements scolaires, Université de Montréal.

Arksey, H. & O'Malley, L. (2005). Scoping Studies: Towards a Methodological Framework. *International Journal of Social Research Methodology, 8*(1), 19-32.

Bero, L.A, Grilli, R., Grimshaw, J.M., Harvey, E., Oxman, A.D., & Thomson, M.A. (1998). Closing the gap between research and practice: an overview of systematic reviews of interventions to promote the implementation of research findings. *British Medical Journal, 317*(7156), 465-468.

Bowen, S. & Martens, P.J. (2005). The Need to Know Team. Demystifying knowledge translation. Learning from the community. *Journal of Health Research and Policy, 10*(4), 203-211.

Centre de liaison sur l'intervention et la prévention psychosociales – CLIPP (2011); http://www.clipp.ca/fr/content/clipp-bref.

Craig, P. Dieppe, P., Macintyre, S., Mitchie, S., Nazareth, I., & Petticrew, M. (2008). Developing and evaluating complex interventions: the new Medical Research Council guidance. *British Medical Journal, 337,* 979-983.

Dagenais, C. (2010). Knowledge Transfer in Community-Based Organizations: A needs assessment study. *Global Journal of Community Pryshcology Practice, 1*(2), 13-130.

Dagenais, C. & Janosz, M. (2008). Étude des besoins des chercheurs de l'université en matière de transfert des connaissances issues de la recherche. Regroupement VINCI, Bureau Recherche- Développement-Valorisation. Université de Montréal.

Dagenais, C., Janosz, M., Abrami, P., Bernard, R., & Lysenko, L. (2008). *Intégrer les informations issues de la recherche aux pratiques professionnelles des enseignants et des gestionnaires dans les écoles : Pour un modèle de transfert des connaissances adapté au domaine de l'éducation*. Rapport « public élargi » préparé pour le Conseil Canadien sur l'Apprentissage, Ottawa, Canada.

Davis, D., Evans, M., Jadad, A., Perrier, L., Rath, D., Ryan, D., et al. (2003). The case for knowledge translation: Shortening the journey from evidence to effect. *British Medical Journal, 327,* 33–35

Davis, P., & Howden-Chapman, P. (1996). Translating research findings into health policy. *Social Science and Medecine, 43*(5), 865-72.

Denis, J.-L., Hébert, Y., Langlay, A., Lozeau, D., & Trottier, L.-H. (2002). Explaining Diffusion Patterns for Complex Health Care Innovations. *Health Care Manage Rev, 27*(3), 60–73.

Denis, J.-L., Lehoux, P., & Champagne, F. (2004). Knowledge utilization in health care: From finetuning dissemination to contexualizing knowledge. In L. Lemieux-Charles & F. Champagne (Eds.), *Using knowledge and evidence in health care: Multidisiplinary perspectives* (pp. 18-40). Toronto: University of Toronto Press.

Dobbins, M., Robeson, P., Ciliska, D., Hanna, S., Cameron, R., O'Mara, L., DeCorby, K., & Mercer, S. (2009). A description of a knowledge broker role implemented as part of a randomized controlled trial evaluating three knowledge translation strategies. *Implementation Science, 4*(23), 1-9.

Dobbins, M., Rosenbaum, P., Plews, N., Law, M., & Fysh, A. (2007). Information transfer: what do decision-makers want and need from researchers? *Implementation Science, 2*(20), 1-12.

Egan, M., Bambra, C., Petticrew, M., & Whtehead, M. (2009). Reviewing evidence on complex social interventions: appraising implementation in systematic reviews of the health effects of organisational-level workplace interventions. *Journal of Epidemiological and Community Health, 63*(1), 4-11.

Estabrooks, C.A., Floyd, J.A., O'Leary, K.A., & Gushta, M. (2003). Individual determinants of research utilization: a systematic review. *Journal of Advanced Nursing, 43*(5), 506–520.

Estabrooks, C.A., Thompson, D.S., Lovely, J.E., & Hofmeyer, A. (2006). A Guide to Knowledge Translation Theory. *The Journal of Continuing Education in the Health Profession, 26*(1), 25-36.

Feldman, S. (1999). Strangers In The Night: Research And Managed Mental Health Care. *Health Affairs, 18*(5), 48-51.

Gagliardi, A. R., Fraser, N., Wright, F. C., Lemieux-Charles, L., & Davis, D. (2008). Fostering knowledge exchange between researchers and decision-makers: Exploring the effectiveness of a mixed-methods approach. *Health Policy, 86*, 53-63.

Gélinas, A., & Pilon, J.-M. (1994). Le transfert des connaissances en recherche sociale et la transformation des pratiques sociales. *Nouvelles pratiques sociales, 7*(2), 75-91.

Golden-Biddle, K., Reay, T., Petz, S., Witt, C., Casebeer, A., Pablo, A., & Hinings, C.R. (2003). Toward a communicative perspective of collaborating in research: the case of the researcher decision-maker partnership. *Journal of Health Services Research and Policy, 8*(S2), 20-5.

Graham, I.D., Logan, J., Harrison, M.B., Straus, S.E., Tetroe, J., Caswell,W., & Robinson, N. (2006). Lost in Knowledge Translation: Time for a Map? *Journal of Continuing Education in the Health Professions, 26*(1), 13-24.

Greenhalgh, T., Robert, G., Bate, P., Kyriakidou, O., Macfarlane, F., & Peacock, R. (2004). *How to Spread Ideas: A Systematic Review of the Literature on Diffusion, Dissemination and Sustainability of Innovations in Health Service Delivery and Organisation*. London: British Medical Journal Publications.

Grol, R. (2001). Successes and failures in the implementation of evidence-based guidelines for clinical practice. *Medical Care, 39*(8), II46-II54.

Grol, R., & Grimshaw, J. (2003). From best evidence to best practice: effective implementation of change in patients'care. *The Lancet, 362*(9391), 1225-1230.

Huberman, M. (1993). Linking the Practitioner and Researcher Communities for School Improvement. *School Effectiveness and School Improvement, 4*(1), 1-16.

Innvaer, S., Vist, G., Trommald, M., & Oxman, A. (2002). Health policymakers'perceptions of their use of evidence: a systematic review. *Journal of Health Services Research and Policy, 7*(4), 239–44

Instituts de recherche en santé du Canada (2011). http://www.irsc-cihr.gc.ca/f/43716.htm.

Institute for Work & Health (2011). *Report on Knowledge Transfer and Exchange Practices: A systematic review of the quality and types of instruments used to assess KTE implementation and impact*. Toronto, Canada.

Jacobson, N., Butterill, D., & Goering, P. (2003). Development of a framework for knowledge translation: Understanding user context. *Journal of Health Services Resources and Policy, 8*(2), 94–99.

Lomas, J. (2000). Connecting Research and Policy. *Isuma: Canadian Journal of Policy Research, 1,* 140–144.

Landry, R., Amara, N., & Laamary, M. (1998). *Utilization of Social Science Research in Canada*. Quebec: University Laval, 1998.

Malo, M., & Robert, E. (2011). Proposition: Canevas pour une Scoping Study. http://recherchevtc.pbworks.com/w/page/39079873/Canevas%20pour%20une%20Scoping%20Study.

McGlynn, E.A., Asch, S.M., Adams, J., Keesey, J., Hicks, J., DeCristofaro, A., & Kerr, E.A. (2003). The Quality of Health Care Delivered to Adults in the United States. *The New Englend Journal of Medecine, 348*(26), 2635-2645.

McKibbon, K.A., Lokker, C., Wilczynski, N.L., Ciliska, D., Dobbins, M., Davis, D.A., et al. (2010). A cross-sectional study of the number and frequency of terms used to refer to knowledge translation in a body of health literature in 2006: a Tower of Babel? *Implementation Science, 5*(16), 1-22.

Nutley, S. (2010). *Evidence-informed practice: Using research to improve services for children and young people*, in Walshe K, Harvey G and Jas P (eds) (2010), Connecting knowledge and performance in public services: from knowing to doing, Cambridge University Press.

Nutley, S., Jung, T., & Walter, I. (2008). The many forms of research-informed practice: a framework for mapping diversity. *Cambridge Journal of Education*, 38(1), 53-71.

Nutley, S., Walter, I., & Davies, H.T.O. (2009). Promoting Evidence-based Practice: Models and Mechanisms From Cross-Sector Review. *Research on Social Work Practice*; 19(5); 552-559.

Ouimet, M., Landry, R., Amara, N., & Belkhodja, O. (2006). What factors induce health care decision-makers to use clinical guidelines? Evidence from provincial health ministries, regional health authorities and hospitals in Canada. *Social Science and Medicine, 62,* 964-976.

Pawson, R., Greenhalgh, T., Harvey, G., & Walshe, K. (2005). Realist review: A new method of systematic review designed for complex policy interventions. *Journal of Health Services Research and Policy, 10(S1),* 21-34.

Pluye, P., Robert, E., Cargo, M., Bartlett, G., O'Cathain, A., Griffiths, F., et al. (2011). Proposal: A mixed methods appraisal tool for systematic mixed studies reviews. http://mixedmethodsappraisaltoolpublic.pbworks.com.

Rich, F. R. (1997). Measuring Knowledge Utilization: Processes and Outcomes. *Knowledge and Policiy: The International Journal of Knowledge Transfer and Utilization, 10*(3), 11-24.

Ridde, V. (2007). Réduire les inégalités sociales de santé : santé publique, santé communautaire ou promotion de la santé ? *Promotion & Education, 14*(2), 111-114.

Société canadienne de psychologie (2000). *Code canadien de déontologie professionnelle des psychologues – Troisième édition.* Ottawa, Canada.

Schuster, M., McGlynn, E., & Brook, RH. (1998). How good is the quality of health care in the United States? *Milbank Quartely, 76*(4), 517-563.

Speller, V., Wimbush, E., & Morgan, A. (2005). Pratique basée sur les données probantes en promotion de la santé : comment faire pour que ça marche. *Promotion & Education, 12*(S1), 46.

Weiss (1998). Have We Learned Anathing New About the Use of Evaluation? *Americain Journal of Evaluation, 19*(1), 21-33.

Wilson, P.M., Petticrew, M., Calnan, M.W., & Nazareth, I. (2010). Disseminating research findings: what should researchers do? A systematic scoping review of conceptual frameworks. *Implementation Science, 5*(91), 1-37.

Woolf, S.H., & Atkins, D. (2001). The Evolving Role of Prevention in Health Care: Contributions of the U.S. Preventive Services Taks Force. *Americain Journal of Preventive Medecine, 20*(S3), 13-20.
